# Artificial Hair Cell Sensor Based on Nanofiber-Reinforced Thin Metal Films

**DOI:** 10.3390/biomimetics9010018

**Published:** 2024-01-02

**Authors:** Sajad A. Moshizi, Christopher J. Pastras, Shuhua Peng, Shuying Wu, Mohsen Asadnia

**Affiliations:** 1 School of Engineering, Macquarie University, Sydney, NSW 2109, Australia; sajad.abolpourmoshizi@mq.edu.au (S.A.M.); christopher.pastras@mq.edu.au (C.J.P.); 2 School of Mechanical and Manufacturing Engineering, University of New South Wales, Sydney, NSW 2052, Australia; shuhua.peng@unsw.edu.au; 3 School of Aerospace, Mechanical and Mechatronic Engineering, The University of Sydney, Sydney, NSW 2006, Australia; shuying.wu@sydney.edu.au

**Keywords:** platinum thin film, carbon nanofibers, piezoresistive sensors, artificial hair cells, vestibular system

## Abstract

Engineering artificial mechanosensory hair cells offers a promising avenue for developing diverse biosensors spanning applications from biomedicine to underwater sensing. Unfortunately, current artificial sensory hair cells do not have the ability to simultaneously achieve ultrahigh sensitivity with low-frequency threshold detection (e.g., 0.1 Hz). This work aimed to solve this gap by developing an artificial sensory hair cell inspired by the vestibular sensory apparatus, which has such functional capabilities. For device characterization and response testing, the sensory unit was inserted in a 3D printed lateral semicircular canal (LSCC) mimicking the environment of the labyrinth. The sensor was fabricated based on platinum (Pt) thin film which was reinforced by carbon nanofibers (CNFs). A Pi-shaped hair cell sensor was created as the sensing element which was tested under various conditions of simulated head motion. Results reveal the hair cell sensor displayed markedly higher sensitivity compared to other reported artificial hair cell sensors (e.g., 21.47 mV Hz^−1^ at 60°) and low frequency detection capability, 0.1 Hz < f < 1.5 Hz. Moreover, like the LSCC hair cells in biology, the fabricated sensor was most sensitive in a given plane of rotational motion, demonstrating features of directional sensitivity.

## 1. Introduction

Mechanosensory hair cells evolved hundreds of millions of years ago in primitive fish to provide accurate sense data for tiny vibrations in the aquatic environment. In extant mammals, mechanosensory hair cells provide the cellular machinery to drive hearing and balance sensations [1,2]. Hair cells are remarkably sensitive biosensors and transduction units capable of achieving a wide frequency bandwidth, dynamic range, and signal detection as small as 20 µPa of pressure and 1 picometre of eardrum displacements at the threshold of human hearing [3,4,5]. These cells convert mechanical energy into electrical information for downstream signal processing. Filamentous hair-like structures called cilia are the fundamental drivers of these mechanosensitive cells that allow gating current to enter the hair cell for resting membrane potential modulation in sensory coding. Promisingly, these cilia have shown to possess robustness with regards to material properties, biomechanics and energy consumption [6,7]. The sensitivity of biological transduction in inner ear hair cells comes from the gating apparatus called mechanoelectrical transduction (MET) channels, its molecular machinery and unique protein conformations. MET channels at the apex of the hair cell are sensitive to nanometer displacements and modulate the gating current (dominated by potassium) via changes in tip link stiffness. Additionally, the inner ear hair cells evolved the remarkable ability to contract and elongate, acting as pistons, to counteract viscous damping in the inner ear fluids, which amplify the mechanical traveling wave of the basilar membrane by ~60 dB or 1000 times [8]. This process is driven by the motor protein, called prestin, located in the cytoskeleton of the hair cells, which behaves as a biological ‘piezo-element’ [9]. Hence, engineering a biosensor using piezo-resistive properties, with a similar topography to inner ear hair cells has potential for low-threshold signal detection of mechanical stimuli and high sensitivity. 

**Figure 1:**
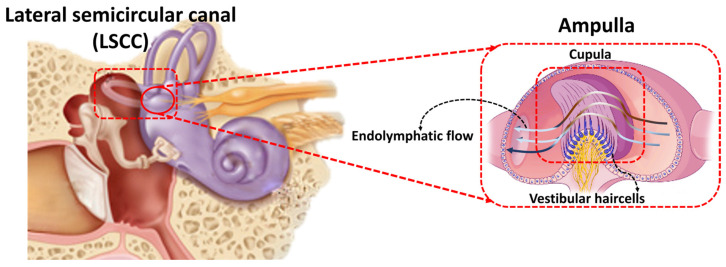
A graphic of the biological inner ear, semicircular canals, and hair cells [11].

With regards to the relevant frequency range of biological hair cells in the inner ear, the vestibular system or the peripheral balance organ, has the ability to detect low-frequency rotations and accelerations. It is not difficult to appreciate why the mammalian vestibular system evolved the ability to detect low-frequency stimuli, as our naturalistic body motion is below 5 Hz, and locomotion in the terrestrial environment, and processing signals or head relative to body movement is referenced to maintained acceleration due to the earths gravitational field (effectively DC). Figure 1 shows a schematic image of the biological semicircular canal and hair cells inside the cupula. Hence, there are obvious advantages of developing an artificial sensory system, based on the features of the vestibular system. The rotational sensor of the vestibular system comprises three interconnected semicircular canals (SCCs) containing the bony and membranous labyrinth, filled with the incompressible Newtonian fluids called perilymph and endolymph, respectively. Hair cells are housed within a gelatinous material called the cupula, which is positioned in the ampulla. Head motion causes the endolymph to flow through the canal ducts, deflecting the cupula and sensory hair cells. One approach to engineer a low-frequency sensory system analogous to the SCCs is to design a similar mechanoelectrical transduction apparatus, ‘the sensor’, which translates motions and mechanical deformations into electrical information and signals. To date there have been considerable efforts to fabricate artificial hair cell sensors with various levels of sensitivity and velocity/frequency detection range. Our group recently developed a hydrogel/graphene-based hair cell sensor with a frequency threshold as low as 0.1 Hz and sensitivity of around 4 mV/Hz [10], while PDMS/graphene-based revealed a sensitivity of 25.33 mV/Hz as low as 0.5 Hz [11]. For detailed information, a comprehensive study has been conducted on bioelectronic devices to interface with the peripheral vestibular system in Ref. [12]. Taking inspiration from sensory hair cells is a well-established technique to fabricate flow sensors for a broad range of applications such as microfluidic devices [13], leakage detection systems [14], navigation systems [15] and respiratory monitoring [16] to measure flow velocity and frequency. 

It is no surprise the elegance and simplicity of the biological transduction apparatus (the hair bundle/cell system) has inspired engineers looking to fabricate sensitive signal detection systems over a range of research fields, from underwater sensing [17], force detection [18], to respiratory flow sensing [19]. However, another promising future application lies in the development of artificial biomimetic hair cells as replacements of damaged inner ear sensory systems in health and disease [12]. In principle, such a device may serve as an inner ear prosthesis. Distinct from current neural implants, like the Cochlear Implant, biomimetic hair cells could replicate dead inner ear hair cells and send synthetic mechanosensory signals to intact nerve terminals with the goal of respectable frequency resolution and sensitivity. However, a limitation with all inner ear neural interfaces is their limited capacity to transmit signal information within the viscous environment of the inner ear. That is, an artificial sensory system could improve sensitivity at the ‘front-end’, but the real challenge is integrating the mechanosensory information from the sensory device with the neurons across the highly resistive extracellular environment, prone to electrical shunting and decreased signal to noise. There may be methods to improve this transfer of information, such as hybrid electrical and optical stimulation, however this may become less effective in the diseased nerve. At the very least, such a technology offers a potential tool for future implants and prosthetic devices of biological sensory systems, which may serve to enhance signal processing, like a hearing aid.

In terms of sensor materials, conductive thin metal films, such as platinum and gold thin films deposited on elastic substrates, have demonstrated potential due to their inherently high conductivity. Moreover, thin metal films can be shaped on micro- and nano-scale profiles using fabrication processes such as electroplating, sputtering, and evaporation [1,20,21,22], which is ideal for future applications requiring miniaturization. When the sensor is subjected to mechanical stretching, long-channel cracks are formed over the thin film. Therefore, the strain sensors made of conductive thin metal films deposited on elastic substrates have ultrahigh gauge factor. In this technique, nanofillers bridge and deflect long-channel cracks and consequently form short microcracks [20]. An attractive approach for bolstering the strain-resilient electrical characteristics of thin metal films involves the incorporation of fiber reinforcement. The application of fiber-reinforced composites has been broadly embraced as a means to enhance both the mechanical and electrical attributes of the polymer matrix [23,24,25,26,27,28,29].

In this work, we developed a hair cell sensor based on Pt thin film reinforced by carbon nanofibers (CNFs). This hair cell sensor utilizes nanofiber-metal composite thin film to develop a miniaturized hair cell sensor with high sensitivity and low-frequency detection threshold. As a potential application of the hair cell sensor, a 3D printed LSCC was equipped with the hair cell sensor and placed at the center of a rotary table capable of replicating human head motion. The whole system was aligned with the slope of 30 degree to mimic the orientation of LSCC in the head. The hair cell sensor was tested for various rotational angles, frequencies, and rotational axes.

## 2. Materials and Methods

**Figure 2:**
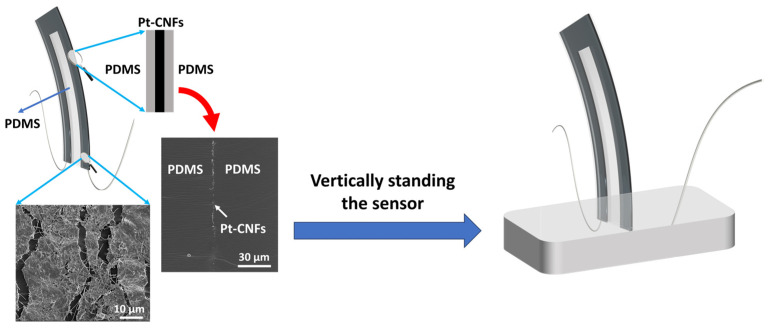
Schematic image of the proposed hair cell sensor with magnified top view of SEM images of Pt-CNFs thin film (30 µm diameter scale bar) and cross-sectional view of Pt-CNFs/PDMS sandwiched layer (10 µm diameter scale bar).

The detailed fabrication of the nanofiber-metal composite (Pt-CNFs/PDMS) has been described elsewhere [20]. Briefly, the proposed hair cell sensor is composed of CNFs coated on a thin PDMS layer and then sputter-coating a thin layer of conductive platinum (Pt). Upon stretching, long cracks are formed in the Pt film, which can be shorted, deflected, and bridged by the CNFs. Indeed, the presence of CNFs strengthened the Pt matrix, leading to a progressive rise in resistance when the sensor underwent stretching. The use of CNF reinforcement effectively redirects and spans microcracks within the Pt phase. As a result, it restricts the average crack length and the spacing between cracks in the Pt/CNFs thin films. The resultant conductive thin film shows steady increase in resistance, which has linear correlation with the stretching strain, enabling accurate measurement and straightforward calibration [20]. The Pt-CNFs/PDMS nanocomposite film was cut into Pi-shape (Figure 2) where copper wires were connected to the two legs with conductive 05063-AB silver paste (SPI Supplies). Another layer of PDMS was added and cured to the top of the sensing element to encapsulate the sensing element and wires’ connections. After that, the sensor was vertically placed inside a piece of Blu-Tack (Bostik, Australia). The schematic of the hair cell sensor with SEM images (cross-sectional and top view) of the Pt-CNFs thin film has been illustrated in Figure 2.

The sensing capabilities of Pt/CNFs sensors exhibited a remarkable enhancement in comparison to the pure Pt coating. The sensing range achieved an ultrahigh sensitivity of 431 and a linearity of 99% [16]. These values surpass those reported for strain sensors based on cracks. Conversely, the strain sensor utilizing a pure CNF layer without Pt coating displayed a considerably lower gauge factor of 15.6 and a sensing range of 61% strain [20].

The main goal in developing the featured flow sensor was to faithfully emulate the low-frequency operational characteristics of semi-circular canal (SCC) vestibular hair cells. These cells are known for reliably detecting subtle changes in endolymph motion within the SCC duct. To evaluate the sensor’s performance under low-frequency rotations, it was intricately integrated into a 3D-printed semicircular canal. This innovative approach aimed to create a controlled environment mirroring the biological conditions of sensory hair cells in the inner ear.

For precise replication of the human lateral SCC (LSCC), a magnetic resonance imaging (MRI) scan of the LSCC was conducted. The MRI scan provided essential insights into the canal’s exact dimensions, serving as a detailed blueprint for subsequent modeling and fabrication using SOLIDWORKS^®^ 2019 software SOLIDWORKS 2019 SP5.0 and a Stereolithography 3D printer. The decision to magnify the 3D-printed LSCC, making it six times larger than the original MRI scan, facilitated practical integration of the sensor within the canal while preserving intricate biological details [10].

Each semicircular canal is most sensitive to a specific rotational axis, and the LSCC, sensitive to yaw-axis rotation, was deliberately chosen. A rotary stage replicated three rotational axes—yaw, pitch, and roll—simulating human head movements. This comprehensive setup allowed for the systematic exploration of the sensor’s response under diverse conditions, including variations in position and oscillation frequency [11,30].

The integration of advanced imaging, 3D printing, and biomechanical simulation underscores the meticulous multidisciplinary approach in evaluating the sensor’s performance. The methodology faithfully replicates the biological conditions within the inner ear, enhancing the relevance and reliability of findings for understanding how biomimetic sensors respond to natural head movements.

Following the preparation of the 3D-printed LSCC, the hair cell sensor was integrated into the canal and placed at the central axis of the rotary stage. To simulate the inner ear environment, the LSCC was filled with deionized water characterized by a density of 1 g mL^−3^ and a viscosity of 1 cP. The experimental setup included a Wheatstone bridge circuit featuring specific resistance values (R_1_ = R_2_ = 815 kΩ, ~R = 680 kΩ), a potentiometer, and an operating voltage (V_in_) of 5.97 V, and a low-noise preamplifier. The DAQ device applied a low-pass filter to optimize the sensor output for analysis.

The stage was set at a 30-degree pitch downward, mimicking the natural inclination of the LSCC within the human head. Controlled conditions were implemented to record sensor responses for three rotational axes and a frequency range from 0.1 Hz to 1.5 Hz. Roll-axis data was excluded due to observed limitations in sensitivity and repeatability, likely stemming from the sensor’s structural characteristics—specifically, its thinness and substantial width, making it less responsive to roll-axis rotations.

## 3. Results and Discussion

**Figure 3:**
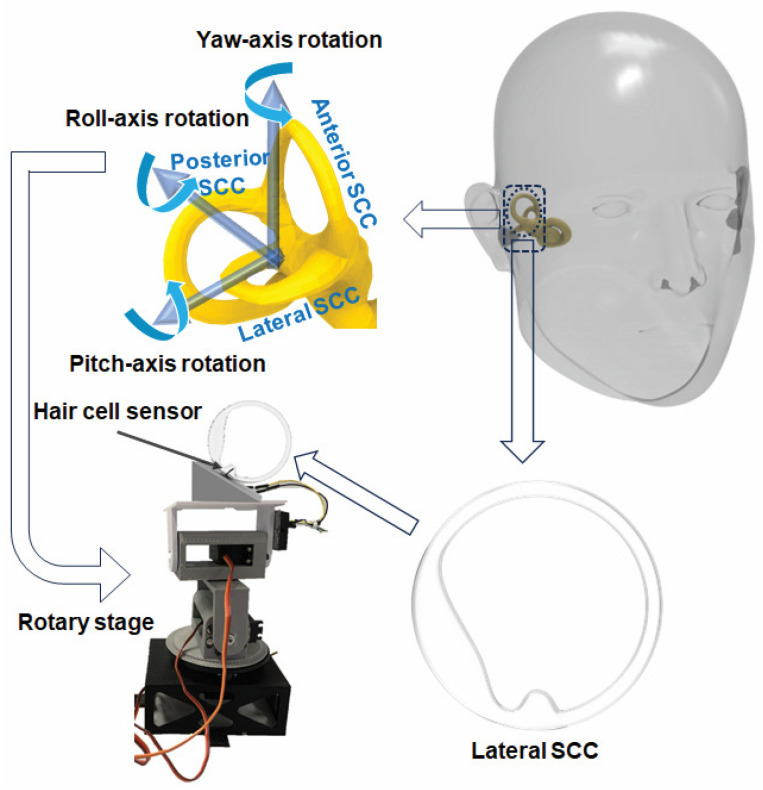
Schematic diagram of the fabrication process of LSCC and experimental setup.

Figure 3 illustrates the sensor response during yaw rotation at a 60° rotational angle across varying frequencies (0.1 Hz, 0.5 Hz, 1 Hz, and 1.5 Hz). The simultaneous recording and plotting of sensor output (blue) and stage position (magenta) underscore the dynamic interplay between the sensor and the fluid dynamics within the LSCC. The frequency sweep at a fixed rotational angle unveils a noticeable increase in sensor output, attributed to heightened fluid momentum. Intriguingly, the sensor’s response manifests two distinct peaks in each cycle—an overarching main peak and a smaller secondary peak. This phenomenon is likely a consequence of the sensor’s non-sinusoidal or distorted motion, potentially induced by uneven pressure distribution resulting from the asymmetric geometry within the LSCC.

**Figure 4:**
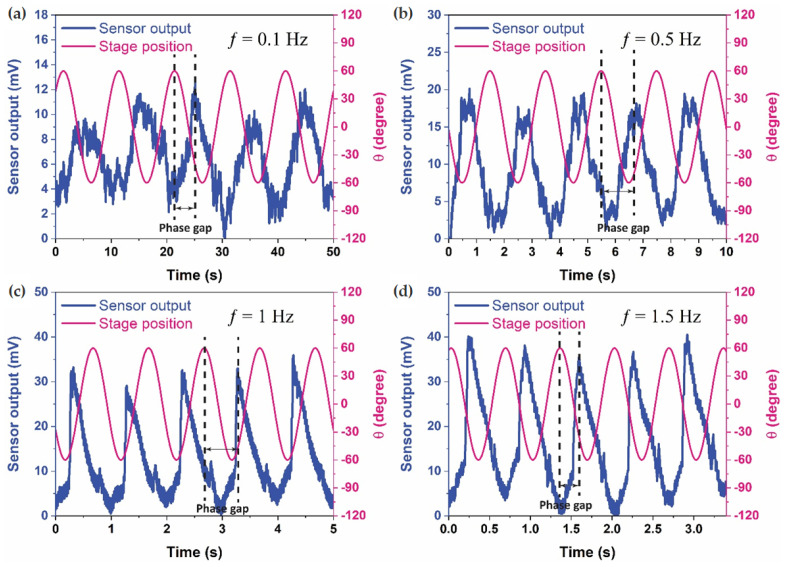
Sensor output and stage position in the time domain for a 60° rotational angle across four frequencies: (**a**) 0.1 Hz, (**b**) 0.5 Hz, (**c**) 1 Hz, and (**d**) 1.5 Hz.

To delve deeper into the dynamics of the system, the phase relationships between continuous stage position and sensor response voltage output signals were meticulously examined. The datasets in Figure 4 reveal repeatable phase lags between the stage position and sensor output, elucidating the presence of inertial lags induced by fluid motion within the LSCC, driven by the motion of the rotary stage. These phase relationships align with prior findings reported in the literature [10,11,30]. 

Notably, the sensor exhibited a clear and robust response, delivering a high voltage output even at the low frequency of 0.1 Hz. However, the Signal-to-Noise ratio exhibited an increase with frequency, reaching its zenith at the highest frequency test of 1.5 Hz. Despite this observation, these findings unequivocally affirm the hair cell sensor’s capability to detect very low frequencies, such as 0.1 Hz, with exceptional sensitivities. The experimental results provide valuable insights into the sensor’s performance under controlled conditions, paving the way for further exploration and potential applications in biomimetic sensory systems.

**Figure 5:**
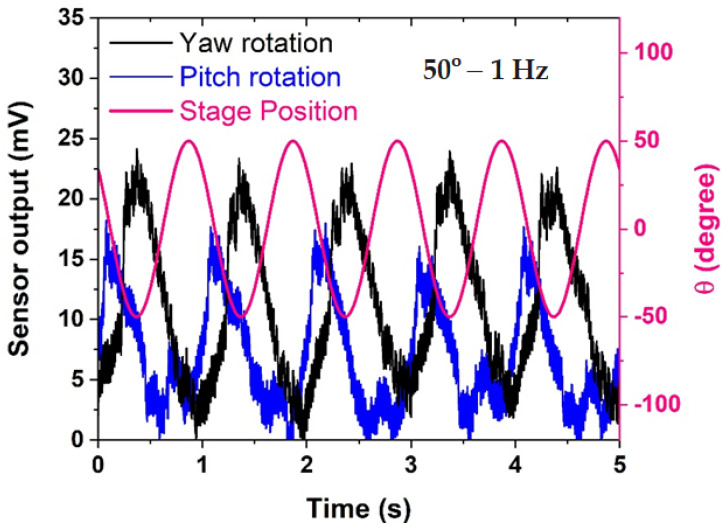
Comparison of sensor response in yaw rotation and pitch rotation versus stage position.

In Figure 5, a detailed analysis of the sensor response during yaw and pitch rotations in comparison to stage position unveils crucial insights into the sensor’s behavior under different rotational conditions. Notably, the sensor exhibits a pronounced preference for detecting yaw rotation, displaying a response that is more than 30% larger compared to pitch rotation. This marked difference in sensitivity between yaw and pitch rotations underscores the sensor’s specialization and heightened efficacy in capturing motions along the yaw axis.

Furthermore, the sensor output during both yaw and pitch rotations reveals the presence of higher-order harmonic components, evident at multiples of the fundamental frequency. This occurrence suggests that the sensor not only faithfully captures the primary rotational movement but also responds to secondary, harmonic oscillations, introducing a nuanced layer of complexity to its output. The manifestation of these harmonics could potentially provide additional information about the dynamic characteristics of the fluid within the LSCC. As predicted, the sensor output shows a marked increase of 28.5% when the LSCC undergoes rotation along the yaw axis, surpassing readings from rotation around the pitch axis. This is attributed to the specific alignment of the LSCC within the same rotation plane as the yaw axis, leading to a more robust induced flow of fluid within the LSCC in comparison to rotation around the pitch axis.

An intriguing observation from Figure 5 is the consistent phase difference between the sensor response and stage position within each cycle for both pitch and yaw rotations. This phase relationship, repeating cyclically, indicates a stable temporal alignment between the sensor’s output and the actual rotational position of the stage. However, it is noteworthy that this phase difference tends to widen when the sensor rotates about the yaw axis. This widening phenomenon suggests a higher fluid inertia in yaw rotation compared to pitch rotation.

The increased phase lag in yaw rotation implies that the fluid dynamics within the LSCC exhibit a more pronounced resistance to changes in motion during yaw movements. This distinction in fluid behavior adds a layer of complexity to the sensor’s response, and it aligns with the inherent characteristics of the LSCC in the human vestibular system. Understanding and quantifying these phase relationships are critical for accurately interpreting the sensor’s output and can provide valuable insights into the biomechanics of the fluid-structure interaction within the artificial semicircular canal.

In summary, Figure 5 not only highlights the sensor’s preference for detecting yaw over pitch rotations but also delves into the nuanced aspects of its response, including the presence of harmonics and the dynamic phase relationships. These findings contribute significantly to comprehending the sensor’s behavior in the context of complex rotational motions, laying the groundwork for refining its applications in biomimetic sensory systems.

**Figure 6:**
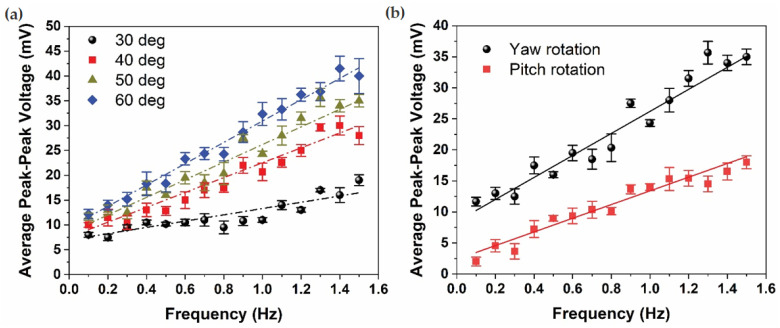
Average peak-peak sensor voltage as a function of frequency under (**a**) four different rotational angles and (**b**) rotational axes, yaw and pitch.

Figure 6 serves as a comprehensive evaluation of the sensor’s sensitivity and performance, systematically exploring the impact of varying frequencies, rotational angles, and rotational axes. In Figure 6a, the influence of increasing rotational angles, specifically 30°, 40°, 50°, and 60°, on sensor sensitivity (expressed in mV/Hz) is depicted. The results reveal a discernible trend, showcasing a proportional increase in sensitivity with greater rotational angles: 6.32 mV/Hz at 30°, 15 mV/Hz at 40°, 17.74 mV/Hz at 50°, and reaching a peak sensitivity of 21.47 mV/Hz at 60°. This correlation suggests that the sensor’s responsiveness amplifies in tandem with the magnitude of the rotational angle, reflecting its capacity to capture and translate intricate rotational motions into electrical signals. In instances of greater frequency and amplitude of rotation, a more pronounced augmentation is evident. This is attributed to an escalation in fluid momentum and secondary flow within the LSCC, resulting in a heightened relative fluid velocity at the sensor. Consequently, this increased fluid motion leads to greater deformation of the sensor [10,11]. 

In Figure 6b, the focus shifts to examining the average peak-peak sensor voltage under both yaw and pitch rotations at a specific rotational angle of 50° across a broad frequency spectrum, ranging from 0.1 Hz to 1.5 Hz. Notably, the sensor sensitivity for pitch rotation is recorded at 10.92 mV/Hz, exhibiting a marginally lower value compared to the sensor sensitivity for yaw rotation, which stands at 17.74 mV/Hz. This disparity in sensitivity between pitch and yaw rotations underscores the sensor’s pronounced inclination towards maximizing its response to yaw rotations, aligning seamlessly with the established biological principles governing the sensitivity of semicircular canals (SCCs). The results affirm that the LSCC, through the integrated sensor, attains its maximum sensitivity when subjected to yaw rotation, reinforcing the biomimetic fidelity of the artificial sensor to its biological counterpart.

In essence, the findings from Figure 6 provide a nuanced understanding of how the sensor’s sensitivity is influenced by varying parameters, offering valuable insights into its performance characteristics. The observed trends not only underscore the adaptability of the sensor to different rotational conditions but also emphasize its specialized responsiveness to specific rotational axes, mirroring the inherent biological principles of the semicircular canals. These results are instrumental in optimizing the sensor’s application in biomimetic systems and contribute to advancing the understanding of artificial sensory devices in the context of complex rotational dynamics.

Table 1 presents a comprehensive comparison of key features between natural biological hair cells and various hair cell sensors developed by Moshizi et al. [10,11,30], shedding light on their performance under different rotational angles (30°, 40°, 50°, and 60°). Each sensor variant offers distinct advantages and trade-offs, contributing to a nuanced understanding of their suitability for specific applications.

The hydrogel/graphene-based hair cell sensor, for instance, stands out for its commendable biocompatibility, aligning well with the natural characteristics of sensory hair cells. This sensor excels in detecting very-low frequencies, reaching down to an impressive 0.1 Hz. However, its sensitivity, quantified at 4 mV/Hz, falls on the lower end of the spectrum compared to other variants.

In stark contrast, the VGNs (Vertical Graphene Nanosheets)/PDMS hair cell sensor showcases an unparalleled ultrahigh sensitivity of 25 mV/Hz. This exceptional sensitivity makes it a formidable contender for applications where minute mechanical stimuli need to be precisely captured. Nevertheless, the trade-off here is a higher frequency detection threshold, pegged at 0.5 Hz. The VGNs sensing element, coated on PDMS with a weight ratio of 25:1, contributes to the sensor’s remarkable sensitivity.

Intriguingly, the Pt-CNFs/PDMS hair cell sensor strikes a balance between sensitivity and frequency detection threshold, presenting a promising middle ground. With a lower frequency detection threshold matching that of the hydrogel/graphene-based sensor (0.1 Hz), the Pt-CNFs/PDMS variant achieves an almost identical sensitivity of 21.4 mV/Hz as the VGNs/PDMS sensor. The Pt-CNFs (carbon nanofibers) sensing element, sandwiched by PDMS with a weight ratio of 10:1, plays a crucial role in this harmonious blend of features.

Drawing inspiration from vestibular hair cells, all the created hair cell sensors represent innovative efforts in replicating the functions of natural biological hair cells. Notably, the Pt-CNFs/PDMS sensor demonstrates performance that resembles that of natural biological hair cells. The ability to maintain a low-frequency detection threshold while rivaling the sensitivity of other high-performance sensors underscores the versatility and applicability of the Pt-CNFs/PDMS hair cell sensor in diverse scenarios. This nuanced evaluation provided by Table 1 contributes significantly to the ongoing efforts in developing biomimetic sensors with tailored features for specific use cases.

## 4. Conclusions

In conclusion, this paper explores the development and performance evaluation of a hair cell sensor inspired by the mechanosensory cells found in the vestibular system of the inner ear. The evolution of mechanosensory hair cells, dating back to primitive fish, serves as the foundation for creating artificial biomimetic sensors. The sensitivity and efficiency of natural hair cells in detecting minute mechanical stimuli have inspired the design of various sensors for applications ranging from underwater sensing to respiratory flow monitoring.

The focus of this study is on a hair cell sensor based on a Pt-CNFs/PDMS nanocomposite, showcasing its potential for replicating the low-frequency operation of semicircular canal vestibular hair cells. The sensor’s design involves a conductive thin film of platinum (Pt) reinforced by carbon nanofibers (CNFs), which, upon stretching, forms cracks that enable accurate measurement of mechanical strain. The fabricated sensor is integrated into a 3D-printed semicircular canal, simulating human head motion, and its performance is evaluated under various conditions.

The results demonstrate the sensor’s ability to detect low-frequency rotations, particularly in the yaw axis, with a sensitivity as high as 21.4 mV/Hz and a frequency threshold as low as 0.1 Hz. The experimental setup, involving a rotary stage and a 3D-printed lateral semicircular canal (LSCC), allows for controlled testing of the sensor’s response to different rotational angles and frequencies. The findings indicate that the sensor’s sensitivity increases with higher rotational angles, showcasing its potential for applications requiring enhanced sensitivity.

Comparison with other hair cell sensors developed by the research group highlights the strengths of the Pt-CNFs/PDMS sensor, particularly its low-frequency detection threshold and high sensitivity. The study also acknowledges challenges, such as limited sensitivity in roll-axis rotations, attributed to the sensor’s thickness and width. Phase relationships between sensor response and stage position, along with the identification of harmonic components, provide insights into the sensor’s behavior under different conditions.

The development of biomimetic hair cell sensors opens up possibilities for future applications, including prosthetic devices for damaged inner ear sensory systems. While the study demonstrates the sensor’s capability to detect low frequencies with high sensitivity, challenges remain in effectively integrating such sensors into the complex and resistive extracellular environment of the inner ear. The paper concludes by emphasizing the potential of this technology for future implants and prosthetic devices, offering improved signal processing akin to a hearing aid. Overall, the presented work contributes valuable insights into the development and application of biomimetic hair cell sensors for a range of scientific and medical purposes.

## Figures and Tables

**Table 1:** Comparison between developed hair cell sensors concerning sensor length, frequency threshold, and sensitivity.

Hair Cell Sensors	Sensor Length (mm)	Frequency Threshold (Hz)	Sensitivity (mV/Hz)			
			30°	40°	50°	60°
LCP-based MEMS [30]	2.7	0.5	0.3	0.7	0.74	0.85
VGNs/PDMS [11]	4.5	0.5	5	8	13	25.3
Hydrogel/graphene [10]	5.5	0.1	2.77	3.98	4.02	3.6
Pt-CNFs/PDMS	4.2	0.1	6.3	15	17.7	21.4
Nature [31]	0.01–0.05	0.001–0.01	30	40	50	60

## Data Availability

Data are contained within the article.
